# Chemical Priming by Isothiocyanates Protects Against Intoxication by Products of the Mustard Oil Bomb

**DOI:** 10.3389/fpls.2020.00887

**Published:** 2020-06-26

**Authors:** Elena Ferber, Julian Gerhards, Miriam Sauer, Markus Krischke, Marcus T. Dittrich, Tobias Müller, Susanne Berger, Agnes Fekete, Martin J. Mueller

**Affiliations:** ^1^Julius-von-Sachs-Institute of Biosciences, Biocenter, Pharmaceutical Biology, University of Würzburg, Würzburg, Germany; ^2^Department of Boinformatics, Biocenter, University of Würzburg, Würzburg, Germany; ^3^Institute of Clinical Biochemistry, University of Würzburg, Würzburg, Germany

**Keywords:** autotoxicity, heat shock response, isothiocyanates, mustard oil bomb, reactive electrophilic species, redox homeostasis, sulforaphane

## Abstract

In Brassicaceae, tissue damage triggers the mustard oil bomb i.e., activates the degradation of glucosinolates by myrosinases leading to a rapid accumulation of isothiocyanates at the site of damage. Isothiocyanates are reactive electrophilic species (RES) known to covalently bind to thiols in proteins and glutathione, a process that is not only toxic to herbivores and microbes but can also cause cell death of healthy plant tissues. Previously, it has been shown that subtoxic isothiocyanate concentrations can induce transcriptional reprogramming in intact plant cells. Glutathione depletion by RES leading to breakdown of the redox potential has been proposed as a central and common RES signal transduction mechanism. Using transcriptome analyses, we show that after exposure of Arabidopsis seedlings (grown in liquid culture) to subtoxic concentrations of sulforaphane hundreds of genes were regulated without depletion of the cellular glutathione pool. Heat shock genes were among the most highly up-regulated genes and this response was found to be dependent on the canonical heat shock factors A1 (HSFA1). HSFA1-deficient plants were more sensitive to isothiocyanates than wild type plants. Moreover, pretreatment of Arabidopsis seedlings with subtoxic concentrations of isothiocyanates increased resistance against exposure to toxic levels of isothiocyanates and, hence, may reduce the autotoxicity of the mustard oil bomb by inducing cell protection mechanisms.

## Introduction

Chemical defense strategies in plants often involve both inducible secondary metabolites, classified as phytoalexins, as well as the constitutive accumulation of secondary defense metabolites, termed phytoanticipins. Since the biological activity of the vast majority of plant secondary metabolites is unspecific and not directed against specific foreign target proteins, plants cells need to protect their own tissues from self-intoxication by their defense chemicals. Besides storage of these compounds in safe compartments, a common strategy is to store biologically inactive secondary metabolite precursors that release biologically active phytochemicals after activation of enzymes triggered by tissue damage, infection or pest attack. An example of this plant defense strategy is the glucosinolate-myrosinase system in Arabidopsis also known as the mustard oil bomb (Shirakawa and Hara-Nishimura, 2018). After tissue damage, however, biologically active phytochemicals can cause cell death and tissue necrosis in plant tissues close to the damage site (Andersson et al., 2015).

Up to 40 biologically inactive glucosinolates, localized in specialized lacticifer-like S-cells in close vicinity to the vasculature and along the leaf margins, have been identified as most abundant phytoanticipins in different Arabidopsis accessions, reviewed in Burow and Halkier (2017). These specialized cells have been shown to contain extremely high levels (>130 mM) of glucosinolates (Koroleva et al., 2010). *Arabidopsis thaliana* Columbia (Col-0) synthesizes aliphatic and indolic glucosinolates in a ratio of about 8:2 in leaves (Brown et al., 2003) and the predominant glucosinolate is the aliphatic glucosinolate glucoraphanin that releases the isothiocyanate (ITC) sulforaphane (SF). In Arabidopsis Col-0, nitriles appear to be major breakdown products besides isothiocyanates, a reaction that is promoted by nitrile-specifier proteins after glucosinolate hydrolysis (Wittstock et al., 2016). However, biological activities are likely associated with the corresponding isothiocyanates.

Glucosinolates can be degraded by the enzymatic activity of myrosinases (ß-thioglucoside glucohydrolases, TGGs) that are localized in guard cells and myrosin phloem cell idioblasts (Thangstad et al., 2004). In Arabidopsis leaves, two redundant myrosinases (TGG1 and TGG2) have been shown to be essential for the breakdown of the predominant aliphatic glucosinolates. In the absence of TGG1 and TGG2, degradation of indolic glucosinolates was also greatly affected (Barth and Jander, 2006).

Tissue damage results in high local production of ITCs. For instance after complete mechanical tissue disruption, about 12 μmol aliphatic and 3 μmol indolic glucosinolates per gram fresh weight (FW) (Barth and Jander, 2006) were degraded within 1 min leading to a corresponding local concentration of glucosinulolate degradation products in damaged tissues of about 15 mM. The toxicity of extracellular or exogenously applied ITCs depends on the structure of the side chain and the way of application (vapor, infiltration, spraying). For instance, exposure to the volatile allyl isothiocyanate (AITC) >0.5 M (Overby et al., 2015b) or infiltration of SF >0.5 mM caused large visible lesions (Andersson et al., 2015) and spraying of different ITCs in the concentration range of 10–100 mM resulted in growth inhibition and leaf bleaching (Hara et al., 2010). However, due to the application mode, the absolute dose of exogenous ITCs taken up by plants is difficult to estimate and to correlate with damage-induced ITC accumulation.

A common feature of ITCs is their lipophilicity and electrophilicity. Due to their high chemical reactivity, exposed thiol groups in glutathione and proteins can be covalently modified. Because of their reactivity, ITCs can be classified as reactive electrophilic species (RES) that display concentration-dependent, broad spectrum toxicity in microorganisms, animals and plants. Toxicity of ITCs also depends on the capacity of cells to detoxify these RES mainly via conjugation to glutathione (GSH). Coupling to GSH can take place as a non-enzymatic reaction or can be catalyzed by an array of glutathione S-transferases (GSTs) (Wagner et al., 2002; Hara et al., 2010) which can be transcriptionally upregulated by ITCs (Overby et al., 2015b).

It has been proposed that the major mode of action of SF in mammalian (Valgimigli and Iori, 2009) as well as in plant cells (Andersson et al., 2015) is to decrease the cellular glutathione pool leading to an increase of the redox potential and eventually to cell death. Since all cell permeable and structurally diverse RES have the concentration-dependent ability to deplete the cellular GSH pool, the resulting increased redox potential could potentially act as a common signal triggering RES-responsive genes.

Indeed, several ITCs (Hara et al., 2010) and structurally different RES oxylipins (Mueller et al., 2008; Muench et al., 2016) have been shown to induce expression of heat shock and detoxification genes at low concentrations while spraying higher concentrations were highly toxic. The magnitude of gene induction and cell death varied when different ITCs were tested. Exposure to AITC vapor has recently been shown to upregulate more than 3900 genes in Arabidopsis seedlings including heat stress, oxidative and general stress as well as detoxification genes (Kissen et al., 2016). However, AITC does not occur in Arabidopsis Col-0 and transcriptome data on Arabidopsis ITCs including SF is not available.

It has been shown that SF both possesses direct antimicrobial properties and, at lower concentrations, acts directly on plant cells to trigger defense responses (Andersson et al., 2015; Schillheim et al., 2018). In addition, we hypothesized that SF and other ITCs produced after herbivore or pathogen attack may also serve as local signals or as metabolic regulators that reduce collateral damage of the mustard oil bomb. We show that ITC – at subtoxic concentrations – trigger cell rescue mechanisms that confer protection against ITC intoxication.

## Results

### Uptake and Metabolism of Exogenous Sulforaphane by Arabidopsis Seedlings

**FIGURE 1:**
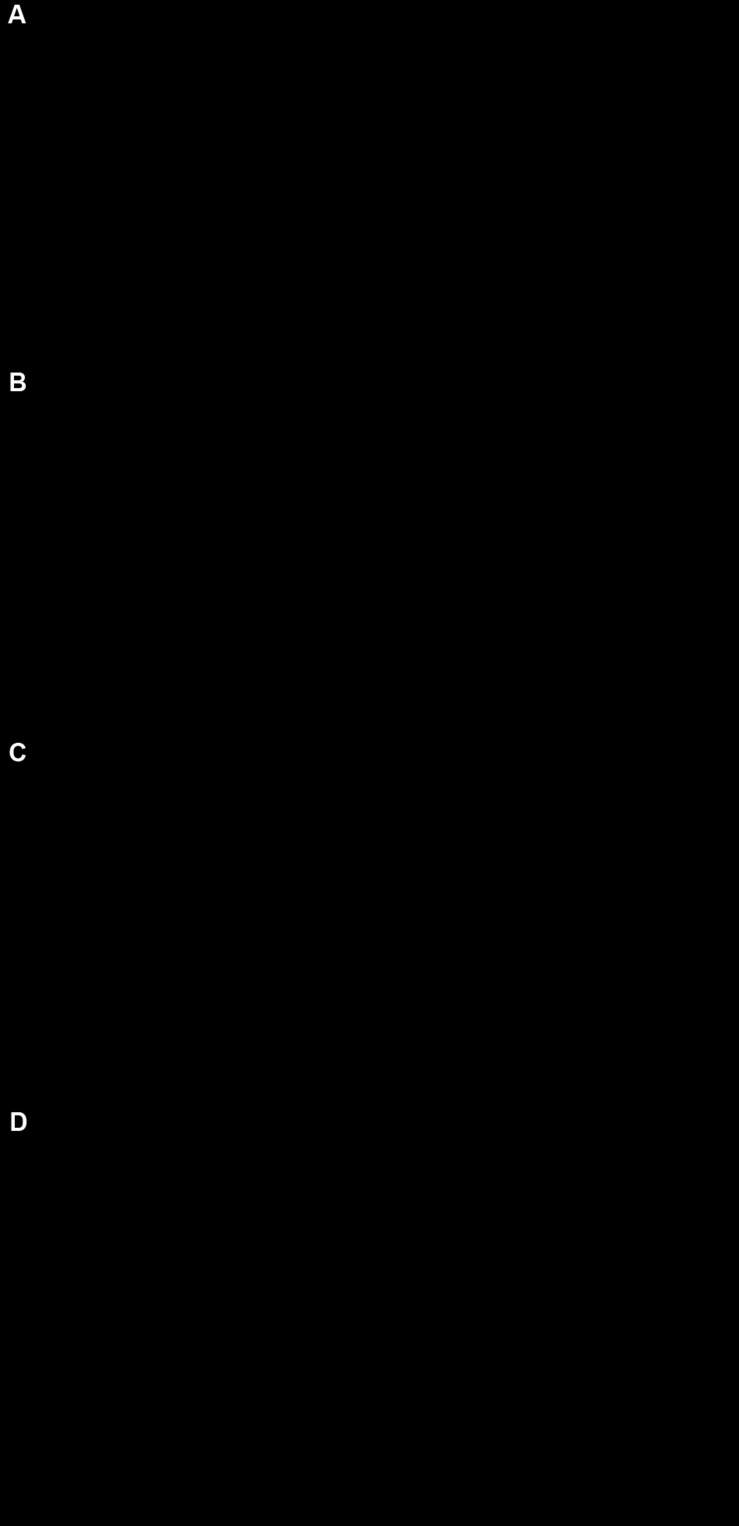
Uptake and metabolism of exogenous sulforaphane. Sulforaphane (50 nmol, final concentration of 100 μM) was applied to 10 Arabidopsis seedlings (123 mg) grown in liquid culture at time zero. The applied dose was 407 nmol/g FW. **(A)** Levels of SF in the medium were determined at the time points indicated in the presence (time 0–4 h) or absence of seedlings (stability control at time 0* and 4* h). **(B–D)** Levels of SF associated with the seedlings **(B)**, levels of SF-GSH in the seedlings **(C)**, and levels of GSH **(D)** were measured 1 h pre-treatment and subsequently for 4 h after the treatments. Data represents means ± SD, *n* = 4 biological replicates within one experiment (each replicate comprised a pool of 10 seedlings).

In order to study the effect of extracellular SF on plant tissues we employed a liquid culture system for Arabidopsis seedlings to which exogenous ITCs and RES could be easily applied for treatment and, after defined exposure times, could be removed for recovery. Previously, ITCs were applied by spraying, leaf inoculation, growth on ITC-containing agar or exposure to isothiocyanate vapors. All these application systems have the disadvantage that the absolute treatment dosage per gram of plant tissue is difficult to estimate. Using the liquid culture system, we first determined uptake of SF by 10 seedlings (123 ± 33 mg, mean ± SD) exposed to 500 μl treatment solution containing 100 μM SF (50 nmol) which corresponds to an absolute dosage of 407 nmol g^–1^ FW. The seedlings were briefly shaken in plain water to remove attached medium prior analysis. As shown in Figure 1, exogenous SF levels rapidly decreased in the culture medium in the presence of seedlings to almost zero during 4 h of incubation. When seedlings were removed from the culture medium prior addition of SF we observed that SF levels (50 nmol, corresponding to 407 nmol g^–1^ FW) remained constant (Figure 1A) indicating that exogenous SF was not degraded or metabolized in the medium. In the presence of seedlings, we observed a strong increase of SF associated with the seedlings (21 nmol g^–1^ FW) immediately after replacing the culture medium with SF-containing medium and subsequently a rapid decrease of seedling-associated SF (Figure 1B). The highest amount of free, seedling-associated SF (21 nmol g^–1^ FW, 5.1% of the applied dose) was determined directly after administration of SF. In parallel to the decrease of SF in the medium and seedlings, rapid metabolism of SF to the glutathione (GSH) conjugate was observed (Figure 1C). The highest amount of SF-GSH conjugate (29 nmol g^–1^ FW, or 7.1% of the applied dose) was determined at the end of the time series (4 h). Potential catabolites of the SF-GSH conjugate such as the cysteinylglycine-, the cysteine, or N-acetlycysteine-conjugate could neither be detected in the seedlings nor in the medium. Since the GSH pool of the seedlings (240 nmol g^–1^ FW) was not significantly depleted by the 407 nmol SF g^–1^ FW treatment (Figure 1D), we conclude that less than 8% of the applied SF was detoxified and stored as GSH conjugate under the low dose SF treatment. Notably, the total GSH in the seedlings (240 nmol g^–1^ FW) would not be sufficient to scavenge the excess SF (407 nmol g^–1^ FW) after uptake.

We performed untargeted metabolite fingerprinting to identify other SF metabolites. Only three low abundant metabolites were found to be significantly increased in the seedlings but not the medium after 4 h: Raphanusamic acid, a known breakdown product of SF (Jeschke et al., 2019) and two unknown metabolites (Supplementary Data, Table S1). Due to their low signal intensities, they appear not to be major SF metabolites. We cannot exclude the possibility that other low molecular weight SF metabolites escaped detection, however, it is likely that SF predominantly binds to proteins as it has been previously been shown to occur in animals (Mi et al., 2007, 2011).

### Transcriptional Response to Sulforaphane, Benzyl Isothiocyanate, and the Oxylipin-RES Prostaglandin A_1_

**FIGURE 2:**
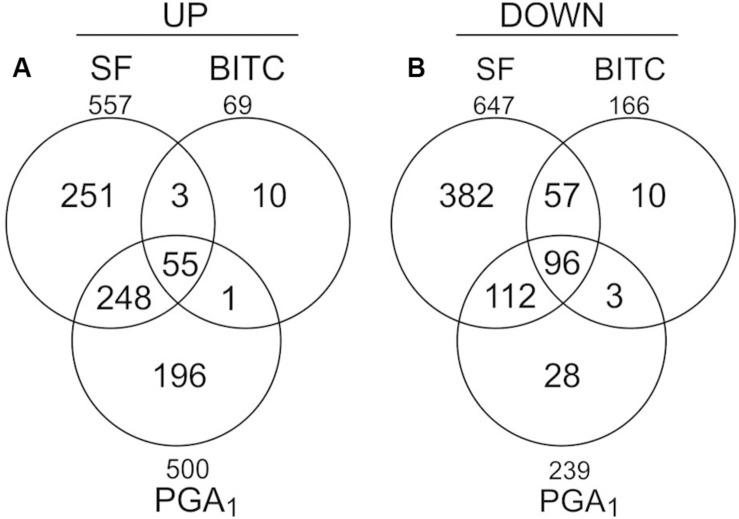
Gene regulation in response to SF, BITC, and PGA_1_ in Arabidopsis seedlings grown in liquid culture. Overlaps of genes up-regulated (fold change ≥ 2), **(A)** or down-regulated (fold change ≤ 0.5), **(B)** in treatments with SF (100 μM), BITC (100 μM), or PGA (75 μM) for 4 h relative to controls.

To analyze the transcriptional response of Arabidopsis seedlings to an exogenous exposure to 100 μM SF in 500 μl we performed genome scale transcriptional profiling by microarray analysis at 4 h (Supplementary Data, Table S2). To compare the transcriptional response to SF with the response to a structural different ITC and a RES-oxylipin, we also performed transcriptional profiling with benzyl isothiocyanate (BITC, 100 μM) and prostaglandin A_1_ (PGA_1_, 75 μM). As shown in Figure 2, SF, BITC and PGA_1_ upregulated 557, 69, and 500 genes, respectively, more than 2-fold at 4 h. Compared to SF, BITC induced considerably fewer genes. However, 84% of the BITC responsive genes were also induced by SF suggesting that BITC triggers a qualitative similar but much weaker response. We observed that PGA_1_, which is structurally different to isothiocyanates, induces 54 and 81% of the genes that are up-regulated by BITC and SF, respectively.

When inspecting the 25 most strongly SF-induced genes (Table 1), 19 genes are involved in the GO biological process “response to heat” (using the PlantGSEA tool; Yi et al., 2013) and 17 of these genes were also found to be upregulated by BITC and PGA_1_. Notably, most of the listed heat responsive genes were also known to be responsive to H_2_O_2_ (Table 1).

**TABLE 1:** List of the 25 most strongly upregulated genes after SF treatment. For comparison, fold-changes (FC) in expression of these genes by BITC and PGA_1_ after 4 h as well as AITC after 9 h (Kissen et al., 2016) are shown.

			SF 100 μM	BITC 100 μM	PGA_1_ 75 μM	AITC vapor	Heat	H_2_O_2_
Locus	Description	Gene name	FC	FC	FC			
AT1G52560	HSP20-like chaperones superfamily protein		168	92	672	1779	•	•
AT1G60750	NAD(P)-linked oxidoreductase superfamily protein		147	3	66	10		
AT4G10250	HSP20-like chaperones superfamily protein	ATHSP22.0	110	136	193	978	•	•
AT3G09640	Ascorbate peroxidase 2	APX1B, APX2	108	20	18	1547	•	•
AT4G27670	Heat shock protein 21	HSP21	98	35	75	3053	•	•
AT3G46230	Heat shock protein 17.4	HSP17.4	68	24	60	960	•	
AT4G25380	Stress-associated protein 10	SAP10	66	4	34	140	•	•
AT5G12030	Heat shock protein 17.6A	HSP17.6A	65	21	66	772	•	•
AT1G53540	HSP20-like chaperones superfamily protein		65	40	76	2798	•	•
AT1G48700	2-oxoglutarate and Fe(II)-dependent oxygenase superfamily protein		56	8	25	18		
AT5G07330	Unknown protein		49	29	15	106	•	•
AT5G12020	17.6 kDa class II heat shock protein	HSP17.6II	35	21	41	1150	•	•
AT4G11393	Defensin-like (DEFL) family protein	DEFL202	29	–	–	–		
AT1G16030	Heat shock protein 70B	HSP70b	27	16	87	155	•	•
AT1G72660	P-loop containing nucleoside triphosphate hydrolases superfamily		22	5	6	261	•	•
AT2G36255	Defensin-like (DEFL) family protein		22	–	–	–		
AT2G32120	Heat-shock protein 70T-2	HSP70T-2	20	4	12	165	•	•
AT1G03070	Bax inhibitor-1 family protein	LFG4	20	4	18	298	•	•
AT1G07500	Unknown protein		19	3	4	181		
AT4G33020	ZIP metal ion transporter family	ZIP9	16	–	–	–		
AT5G53680	RNA-binding (RRM/RBD/RNP motifs) family protein		15	–	–	71	•	•
AT1G74310	Heat shock protein 101	HSP101	15	6	27	59	•	•
AT1G71000	Chaperone DnaJ-domain superfamily protein		15	–	–	408	•	•
AT4G21320	Aldolase-type TIM barrel family protein	HSA32	14	9	9	102	•	•
AT2G26150	Heat shock transcription factor A2	HSFA2	14	2	9	719	•	•

**SF, BITC, and PGA_1_ were dissolved into the liquid growth medium of the seedlings while AITC was applied to seedlings grown on petri dishes as a vapor (from a 50 mM AITC solution in vegetable oil). Only FC values determined with an adjusted p ≤ 0.05 were considered. Genes that are involved in the GO biological processes “response to heat” and “response to H_2_O_2_*” *using the PlantGSEA tool (Yi et al., 2013) are indicated with a black dot.**

Previously it has been shown that genes responsive to heat were strongly induced through HSFA1 by structurally different RES species such as the oxylipins 12-oxo-phytodienoic acid and PGA_1_ (Muench et al., 2016) as well as 2-hexenal (Yamauchi et al., 2015). HSFs/HSPs have also been found to belong to the most highly induced genes at 9 h treatment with AITC vapor (Kissen et al., 2016). Although the AITC treatment dose and the exposure time were very different to our experimental conditions, we added the published AITC data to Table 1.

Out of the most strongly SF-induced genes, 22 genes (88%, including all heat responsive genes) were also induced by AITC. Notably, induction of heat responsive genes by AITC was often more than 10-fold stronger than after treatment with SF, BITC, or PGA_1_ (Table 1) which might be due to structural differences, higher dosage or longer exposure time. Stronger response to AITC is also reflected by the absolute number of genes induced after 9 h AITC vapor treatment (2352 genes) compared to 407 nmol g^–1^ FW SF treatment for 4 h (557 genes) (Supplementary Data, Figure S1). The core set of genes induced by SF, BITC, PGA_1_, and AITC included 42 genes of which 28 (67%) were heat responsive genes (Supplementary Data, Table S3). Hence, induction of heat shock responsive genes appears to be common and most strongly affected after treatment with structurally different RES. However, a considerable number of genes was not induced by any of the other RES-treatments: SF (125 genes, 22%), BITC (10 genes, 14%), PGA_1_ (143 genes, 28%) and AITC (1970 genes, 85%). This suggests that structurally different RES induce a set of common genes but also genes that are RES-specific and dependent on the structure (Supplementary Data, Figure S1).

### Wound-Induced Glucosinolate Breakdown Triggers Gene Induction

**FIGURE 3:**
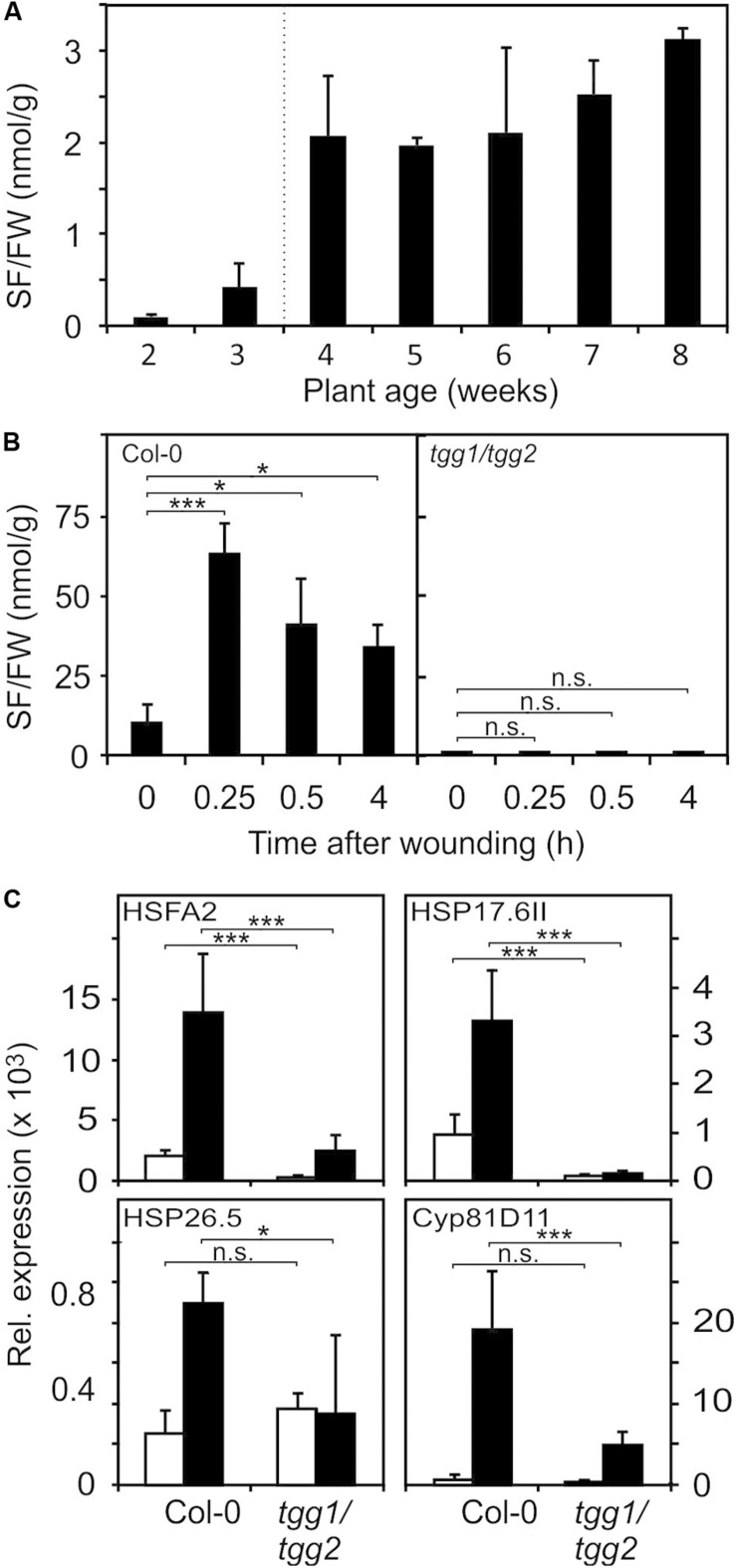
Endogenous SF levels and ITC-induced gene expression. **(A)** SF levels were determined in seedlings (each sample comprised a pool of 10 seedlings) grown in liquid MS medium (2 and 3 weeks) or leaves of plants grown on soil (4–8 weeks), data represent means ± SD, *n* = 3 biological replicates within one experiment. **(B)** Increase of SF levels in leaves of 7-week old wild-type and *tgg1/tgg2* plants grown on soil after wounding, data represent means ± SD, *n* = 4 plants. Time 0 is a no treatment control (leaves were collected and immediately shock frozen). Wounded leaves were collected from the plants after wounding at the times indicated. **(C)** Induction of RES-inducible marker genes in leaves of 6-week old wild-type and *tgg1/tgg2* plants after wounding. Gene expression was determined by RT-qPCR after 0.5 h (HSFA2, HSP17.6II, and HSP26.5) or 4 h (Cyp81D11). Values were normalized to *AtActin2/8*, data represent means ± SD, *n* = 3–6 plants. Asterisks indicate *p* ≤ 0.05 (*), and ≤ 0.005 (***).

A major glucosinolate breakdown product in Arabidopsis Col-0 leaves besides nitriles is SF (Wittstock et al., 2016). Since exogenously administered SF can act as a signal at low concentrations and is toxic at high concentrations (Andersson et al., 2015) we first determined endogenous SF levels in Arabidopsis seedlings and plants. As shown in Figure 3A, we could detect SF in apparently undamaged seedlings and plants grown under optimal conditions. Endogenous levels of SF were around 0.5 nmol g^–1^ FW in seedlings cultured in liquid medium and up to approximately 3 nmol g^–1^ FW in leaves of soil-grown plants (Figure 3A). To determine endogenous SF levels after partial leaf damage by wounding, leaves from 6-week old plants were wounded three times with forceps over the leaf lamina. This treatment led to an accumulation of SF of up to 50 nmol g^–1^ FW corresponding to an average concentration in the wounded leaves of about 50 μM (Figure 3B) in wild type plants. Only a small part of the leaf was directly damaged while SF levels were measured in the whole leave. As expected, the myrosinase deficient double mutant *tgg1 tgg2* was not able to accumulate SF. Notably, SF is expected to accumulate at the wound site close to the midrib and, hence, the SF concentration near the midrib likely reaches much higher concentrations than the levels determined in extracts from whole leaves.

To test whether wound-induced glucosinolate breakdown products induce transcription in wounded leaves, we determined the expression of genes known to be induced by SF and AITC as well as many RES species (Mueller et al., 2008; Kissen et al., 2016) in wild type and *tgg1 tgg2* leaves. Relative expression of HSFA2, HSP17.6II, and HSP26.5 was determined 0.5 h after wounding since these genes display a rapid and transient expression (Muench et al., 2016). Relative expression of most other genes including Cyp81D11 is often highest between 2 and 4 h after treatment. We therefore measured expression of Cyp81D11 4 h after wounding (Figure 3C). Wound-induction of the heat shock marker genes was abolished or strongly reduced in the ITC-deficient *tgg1 tgg2* line, indicating that wound-induced endogenous glucosinolate breakdown products, most likely SF and other ITCs, act as signals that modulate gene expression in wounded leaves. Interestingly, expression of the RES- and jasmonate-inducible Cyp81D11 gene was also markedly reduced in the *tgg1 tgg2* line although the jasmonate pathway is intact in this line suggesting that more than one signal can regulate that gene after wounding.

### Sulforaphane Strongly Induces Heat Shock Response Marker Genes Through HSFA1

**FIGURE 4:**
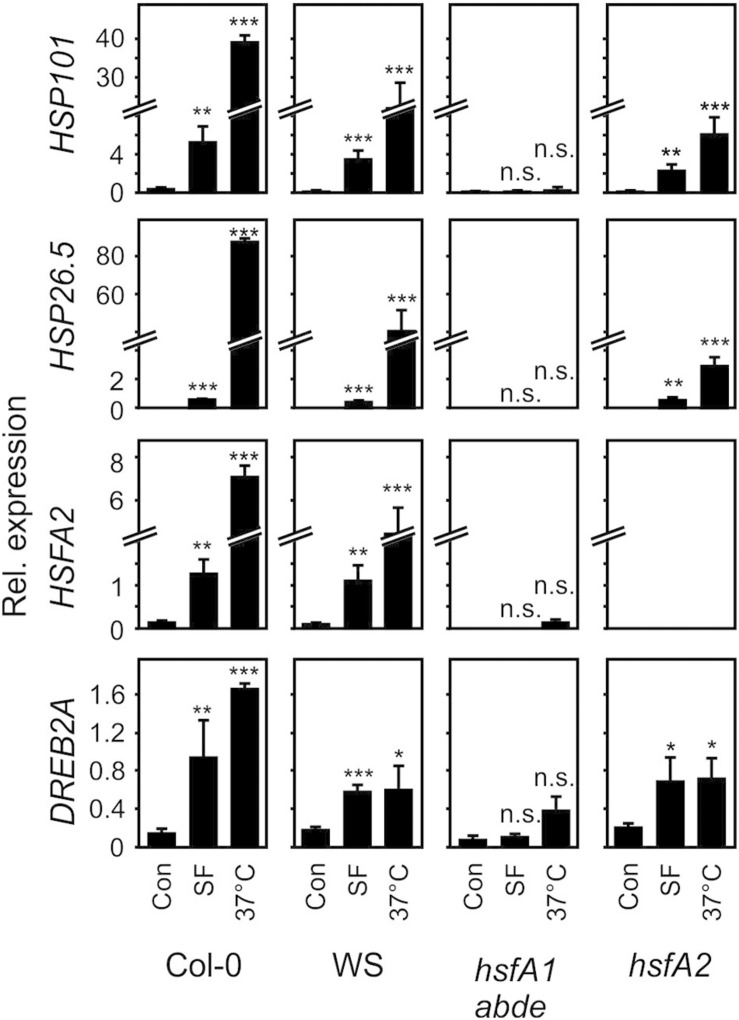
Induction of heat-responsive genes by SF and moderate heat in wild-type (Col-0, WS), *hsfA1 abde*, and *hsfA2* plants. Seedlings were treated with SF (100 μM, corresponding to a treatment dose of 407 nmol/g FW) or moderate heat (37°C) for 4 h and gene expression was analyzed by RT-qPCR. Values were normalized to *AtSAND.* Data represent means ± SD, *n* = 3 biological replicates within one experiment (each replicate comprised a pool of 10 seedlings). Asterisks indicate *p* ≤ 0.05 (*), ≤ 0.01 (**), and ≤ 0.005 (***).

Induction of most heat-responsive genes including the inducible transcription factors *HSFA2* and *dehydration-responsive element-binding protein 2A* (*DREB2A*) as well as *HSP101* and *HSP26.5* is dependent on the four constitutive HSFA1 transcription factors (a, b, d, and e). To compare the activation of these marker genes by moderate heat and SF, we treated seedlings with moderate heat (37°C) or SF (100 μM, corresponding to a treatment dose of 407 nmol g^–1^ FW) for 4 h. The control sample did not receive methanol. We previously tested the effect of different solvents on heat shock gene expression when studying reactive electrophiles dissolved in methanol or DMSO (Muench et al., 2016). Methanol (1%) had no significant effect on gene expression of the selected genes. In addition, we tested expression of these genes in the *hsfA1 abde* quadruple and *hsfA2* mutant plants. Since the *hsfA1* quadruple mutant was generated by crossing single mutants from the ecotype backgrounds Col-0 and Wassilewskija (WS), we used both wild types as controls (Figure 4).

In the wild types Col-0 and WS, SF-induced expression of *HSFA2*, *HSP101*, and *HSP26.5* was about 2–30% of the expression of these genes after 37°C for 4 h. Notably, HSP101 which has been shown to be essential and sufficient to establish acquired thermotolerance was strongly induced at 37°C (68-fold) and SF (15-fold) in *A. thaliana* Col-0 seedlings. The expression of the *DREB2A* gene is induced by heat shock and dehydration via *HSFA1*-dependent and -independent signaling pathways, respectively (Yoshida et al., 2011). In contrast to the other marker genes, induction of *DREB2A* by SF was more similar to the 37°C treatment in the wild types.

SF-induced expression of *HSFA2*, *HSP101*, *HSP26.5*, and *DREB2A* was strictly dependent on HSFA1 master regulator genes of the heat shock response. In addition, the inducible transcriptions factor HSFA2 was not found to be required for up-regulation of *HSP101, HSP26.5* and *DREB2A* by SF. However, HSFA2 was required for the full up-regulation of the small *HSP26.5* gene by heat. Hence, these results indicate that HSFA1s but not HSFA2 are essential for the induction of heat-responsive HSPs by SF.

### Glutathione Depletion and Glutathione Redox Potential Alterations Are Not Involved in the Regulation of the Transcriptional Response to Subtoxic Concentrations of Sulforaphane, Benzyl Isothiocyanate, and Prostaglandin A_1_

**FIGURE 5:**
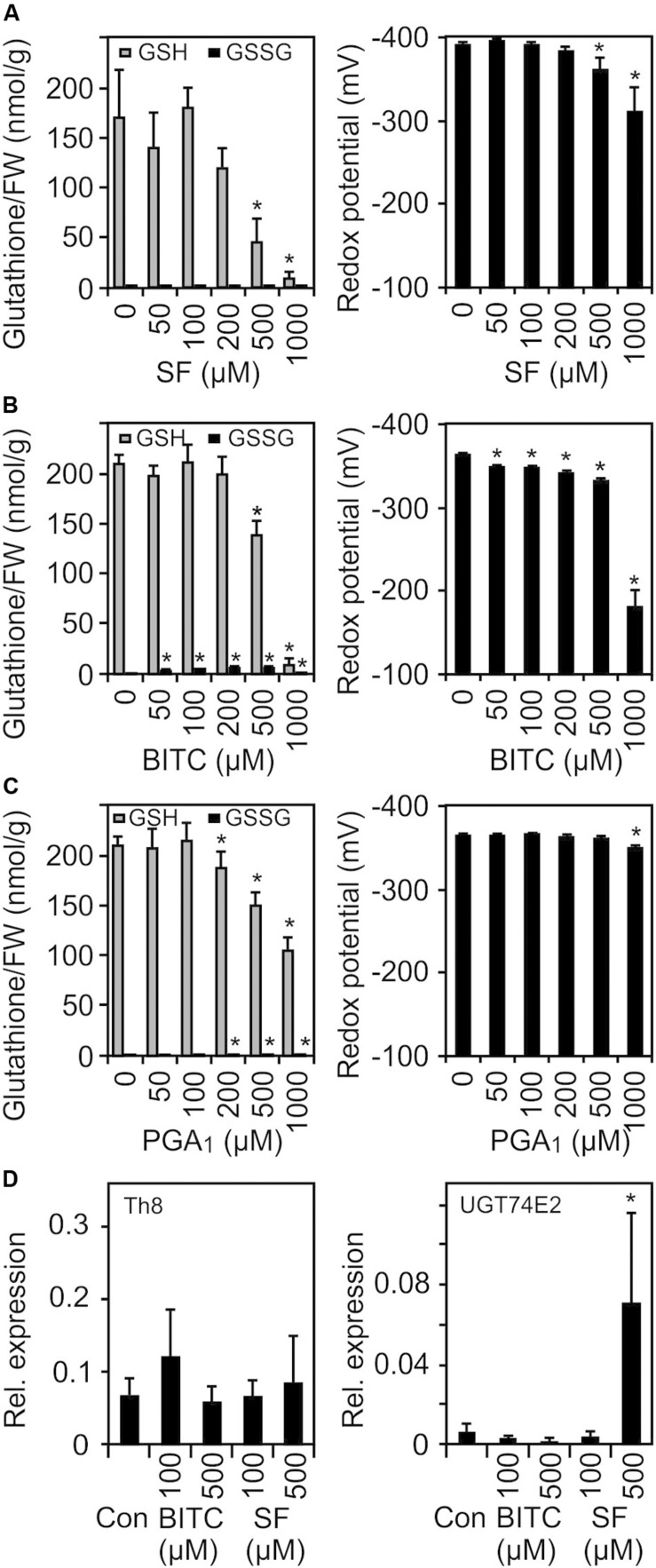
SF, BITC, and PGA_1_ treatments cause oxidation of the cellular glutathione pool and change of the cellular redox potential. Wild-type *Arabidopsis* seedlings grown in liquid culture in MS medium were treated with SF **(A)**, BITC **(B)**, and PGA_1_ **(C)** at the indicated concentrations. Reduced (GSH) and oxidized glutathione (GSSG) contents per gram fresh weight (FW) were measured 4 h after treatment, and the redox potential was calculated. Data represent means ± SD, *n* = 3 biological replicates within one experiment (each replicate comprised a pool of 10 seedlings) **(D)** Expression of low GSH marker genes was determined by RT-qPCR 4 h after treatments with solvent (Con), SF or BITC. Values were normalized to the expression of *AtActin2/8.* Data represent means ± SD, *n* = 4–6 biological replicates within one experiment (each replicate comprised a pool of 10 seedlings). Asterisks indicate *p* ≤ 0.05 (*).

Due to their thiol reactivity, RES can deplete the cellular GSH pool in a concentration dependent manner. Thereby, the redox potential could be increased and sensed as a common signal to triggering RES-responsive genes. To test this hypothesis, we treated 10 day-old Arabidopsis seedlings with SF, BITC and PGA_1_ under treatment conditions similar to the microarray experiments and determined the GSH and the oxidized GSH (GSSG) concentration of the seedlings 4 h after the treatment. As shown in Figure 5, we observed a concentration-dependent decline of the endogenous GSH concentration. In addition, we calculated the GSH/GSSG redox potential (Queval and Noctor, 2007) from the measured endogenous GSH and GSSG levels 4 h after the treatment. However, at concentrations (75–100 μM) that triggered gene regulation, we did not observe a significant decrease of GSH or a change of the calculated redox potential.

The transcriptional response to low GSH content associated with increased redox potential has previously been determined both in GSH-deficient *root meristemless 1-1* (*rml1-1*) seedlings (Schnaubelt et al., 2015) as well as in seedlings that have been treated with the GSH-synthesis inhibitor buthionine sulfoximine (BSO) (Koprivova et al., 2010). When comparing our list of SF-induced genes with the published microarray data of these studies, only 13% of the SF-induced genes were found to be up-regulated in *rml1-1* or BSO-treated seedlings. We also analyzed the expression of two marker genes, a cytosolic h-type thioredoxin (TRX, TH8) and a glucosyltransferase (UGT74E2), that were found to be strongly up-regulated in seedlings with low GSH content. However, both genes were not found to be up-regulated at the concentration used in our microarray experiment. Moreover, heat shock genes are the most strongly up-regulated genes after ITC treatment while these genes appeared not to be regulated after BSO treatment (Koprivova et al., 2010) or were even down-regulated in *rml1-1* seedlings (Schnaubelt et al., 2015). Hence, we conclude that signaling by low glutathione content is not involved in the gene regulation in response to low dose ITC treatment.

### Arabidopsis HSFA1 Transcription Factors Are Important for Resistance Against Isothiocyanate Intoxication

**FIGURE 6:**
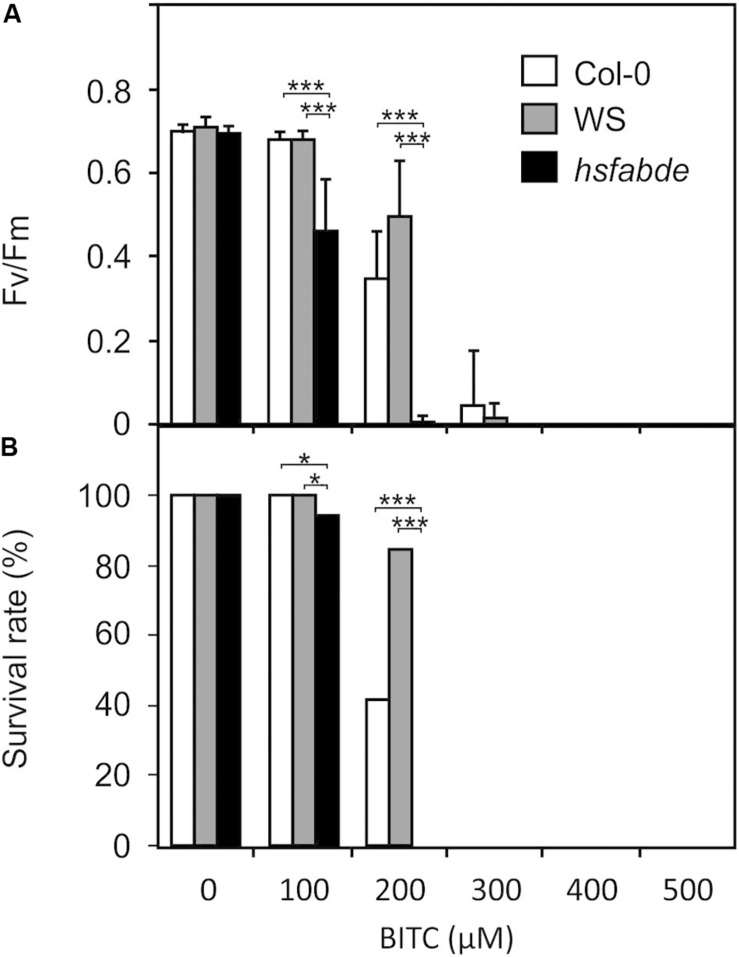
Toxicity of BITC treatments in wild-type and *hsfA1 abde* Arabidopsis seedlings. Wild-type (Col-0, white bars; WS, gray bars) and *hsfA1 abde* (black bars) seedlings were grown in liquid culture in MS medium and treated with BITC at the concentrations indicated for 2 h. Thereafter, the BITC treatment solution was replaced by MS medium for recovery. The effect of BITC treatment on photosynthesis (*F*_*v*_/*F*_*m*_) was determined after 4 h of recovery. Data represent means ± SD, *n* = 8 biological replicates within one experiment and repeated two times with similar results. **(A)** Survival rates **(B)** were determined 7 days after the treatments; statistical analysis was performed on seedlings using the χ^2^ test with *n* ≥ 55 seedlings. Asterisks indicate *p* ≤ 0.05 (*), and ≤ 0.005 (***). The experiment was repeated three times with similar results.

ITCs strongly up-regulate heat shock proteins, a process that could be important to maintain protein integrity under chemical stress. To test this hypothesis, we compared the resilience of wild type (Col-0 and WS) and *hsfA1 abde* seedlings toward BITC intoxication. We observed, that in the liquid culture system, 100 μM BITC was well tolerated by wild type seedlings while photosynthetic efficiency (F_*v*_/F_*m*_) and the survival rate decreased after treatment with BITC concentrations around 200 μM or higher. Moreover, leaf bleaching started to increase at concentrations higher than 200 μM. In contrast to the wild type lines, we observed that in *hsfA1 abde* seedlings F_*v*_/F_*m*_ as wells as the survival rate already decreased after exposure to 100 μM BITC. Photosynthetic efficiency collapsed almost completely and no plants survived the 200 μM treatment (as indicated by complete bleaching of the cotelydons and the first true leaves) indicating a much higher sensitivity of the HSFA1-deficient seedling toward BITC intoxication (Figure 6).

### Chemical Priming by Sulforaphane and Benzyl Isothiocyanate but Not Heat Acclimation Confers Protection Against Isothiocyanate Intoxication

To further test if induction of heat shock genes in wild type seedlings is sufficient to protect against BITC intoxication we heat-acclimated wild type seedlings at 37°C for 2 h. After 2 h of recovery at 22°C, seedlings were exposed to different concentrations of BITC for 2 h. As shown in Figure 4, a 37°C pre-treatment induced a much stronger expression of HSPs than a SF pretreatment. However, we found that strong induction of heat shock genes in seedlings after heat acclimation did not increase the BITC tolerance (Supplementary Data, Figure S2). Hence, rapid HSFA1-mediated up-regulation of HSP by ITC in the wild type may be required and already sufficient to confer some resistance against BITC intoxication. Alternatively, the higher BITC-sensitivity of the *hsfA1 abde* seedlings could be due to a constitutive lower expression of HSFA1-regulated genes. It has been shown that expression of 310 genes was decreased (>2-fold) in the *hsfA1 abde* mutant compared to the wild type in non-stress conditions, and many of them were heat-inducible genes (Yoshida et al., 2011).

**FIGURE 7:**
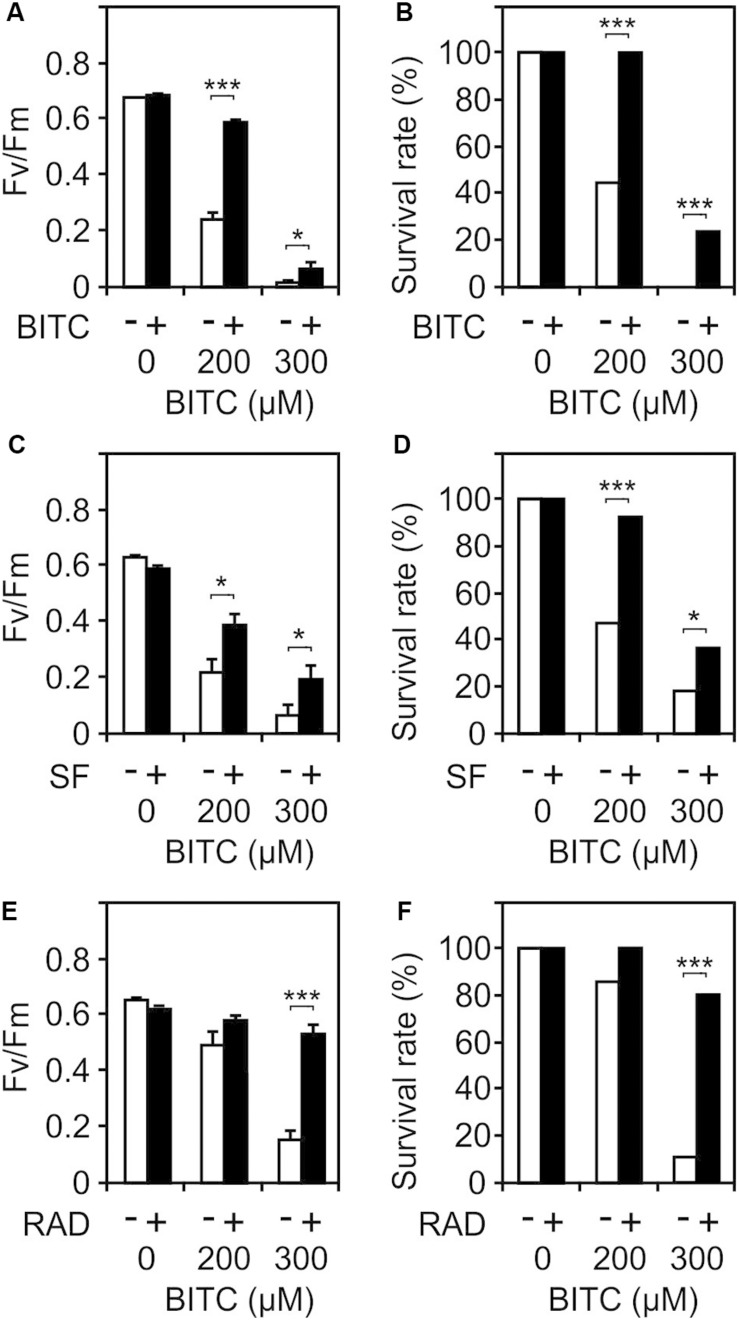
Chemical priming induces resistance toward BITC. *Arabidopsis* seedlings grown in liquid culture in MS medium were pretreated with solvent control (white bars) or different RES: 100 μM BITC **(A,B)**, 100 μM SF **(C,D)**, or 50 μM radicicol **(E,F)** for 24 h (black bars). Thereafter, seedlings were treated with BITC at the concentration indicated for 2 h and, subsequently, allowed to recover in MS medium. The effect of the treatment combination on photosynthesis (*F*_*v*_/*F*_*m*_) was determined after 4 h of recovery. Data represent means ± SE, *n* = 12 biological replicates within one experiment (each comprising 10 seedlings) **(A,C,E)**. Survival rates **(B,D,F)** were determined 7 days after the treatments; statistical analysis was performed on seedlings using the χ^2^ test with *n* ≥53 seedlings. Asterisks indicate *p* ≤ 0.05 (*) and ≤ 0.005 (***). Experiments **(A–F)** were repeated two times with similar results.

Besides heat responsive genes, ITCs induce a great variety of other genes including GSTs that potentially could protect against autotoxicity. To test this hypothesis, we pretreated wild type seedlings with a non-toxic concentration (100 μM) of SF or BITC for 24 h prior to application of toxic concentrations of BITC (200 and 300 μM). As shown in Figures 7A–D, photosynthetic efficiency as well as the survival rates of the SF- or BITC-primed seedlings after intoxication with the model ITC BITC was much higher compared with naïve plants. We also tested radicicol (50 μM) as a chemical priming agents and found that it protected against BITC intoxication (Figures 7E,F). Radicicol has been well studied as a HSP90 inhibitor that strongly induces heat shock genes and increases thermotolerance in Arabidopsis (Yamada et al., 2007). However, radicicol can also be classified as an RES. In fact, microarray analyses revealed that the majority of radicicol-induced genes (73%) was not induced by heat (Yamada et al., 2007). Hence, priming by ITC and potentially other thiol reactive RES can confer protection against ITC to reduce their autotoxicity.

## Discussion

The myrosinase-glucosinolate system is a powerful defense system of Brassicales plants. S-cells in the vasculature of leaves and along leaf margins can accumulate up to 130 mM glucosinolates (Nintemann et al., 2018) which in contact with myrosinases can potentially produce local concentrations of ITCs that are at least 2 orders of magnitude higher than the toxic concentrations for microorganisms (Dufour et al., 2015) and plant cells (Andersson et al., 2015). To ensure safe storage of the ITC precursors, myrosinases are separated from glucosinolates in different specialized cells. We cannot completely exclude the possibility that SF levels (Figure 3) determined in intact tissues reflect an artifact such as an extremely rapid wound response during sampling or release of SF from glutathione- or protein-conjugates during extraction. Alternatively, the low levels of free SF (2–5 nmol g^–1^ FW in mature leaves of undamaged Arabidopsis plants) could be a result of a basal glucoraphanin turnover which potentially could also explain the formation of raphanusamic acid (Jeschke et al., 2019). Since SF is rapidly metabolized (Figure 1), this scenario would imply a slim but continuous degradation of glucoraphanine in intact tissues.

Herbivore feeding, wounding or pathogenic microorganisms that induce a hypersensitive response break S-cells and myrosinase cells open resulting in release of ITCs into the extracellular space around lesions. For instance, wounding (Figure 3) or *Pseudomonas syringae* pv *tomato* DC3000 infection (Andersson et al., 2015) triggered a total SF release of 50–170 nmol g^–1^ FW. SF levels remained elevated for several hours both after wounding (Figure 3) and pathogen infection (Andersson et al., 2015) indicating that the Arabidopsis SF detoxification capacity is not sufficient to remove the high damage-induced local amounts of SF immediately. High local, cytotoxic SF concentrations likely become diluted to subtoxic SF concentrations by radial diffusion into unaffected tissues.

Undamaged tissues of seedlings (10 seedlings, 123 mg FW) grown in liquid culture almost completely absorbed exogenous SF (50 nmol) from the exogenous medium after 2–4 h (Figure 1). The total GSH content in the seedlings (30 nmol) was not significantly decreased after treatment of the seedlings with a low non-toxic SF dose (100 μM solution corresponding to an absolute dose of 50 nmol) but was strongly decreased after treatment with cytotoxic concentrations higher than 200 μM (Figures 1, 5). The predominant SF metabolite that could be detected in the seedlings but not in the medium was the SF-GSH conjugate. However, the major SF metabolite *in planta* is unlikely the GSH conjugate because the endogenous formation of GSH GSH pool is not significantly affected by low concentrations of SF and is by far not sufficient to absorb the excess SF upon treatment after exposure to higher concentrations. To check the possibility of *de novo* GSH formation to compensate for GSH consumption, we checked expression of the two key GSH forming enzymes (glutamate-cysteine ligase (*GSH1*, AT4G23100), glutathione synthetase (*GSH2*, AT5G27380) and all five cysteine forming enzymes of the serine acetyltransferase (SAT) gene family: *SERAT1;1* (At5g56760, SAT-c), *SERAT2;1* (At1g55920, SAT-p), *SERAT2;2* (At3g13110, SAT-m), *SERAT3;1* (At2g17640, SAT-106) and *SERAT3;2* (At4g35640). Except for *SERAT2;1* which was found to be induced by 1.6-fold by SF 4 h after treatment, none of these genes appeared to be regulated by SF in our microarray experiments. We therefore assume that the GSH pool did not respond to the treatment.

Depletion of the endogenous GSH pool associated with an increase of the redox potential has been suggested as a potential trigger of redox-regulated genes. However, exogenous application of subtoxic concentrations of SF, BITC and PGA_1_ that were sufficient to induce transcriptional reprogramming (Figure 2 and Supplementary Data, Table S2) did not induce a marked change in GSH and the calculated GSH redox potential. Hence, GSH depletion is not a relevant signaling mechanism after exposure to low, subtoxic ITC doses while treatment with cytotoxic ITC concentrations potentially triggers low GSH responsive genes (Koprivova et al., 2010) and inhibits growth (Urbancsok et al., 2018). Plants with low GSH levels have been shown to be more sensitive to SF or AITC treatment suggesting that severe GSH depletion leads to cell death (Urbancsok et al., 2018).

Application of ^14^C-labeled SF to animal cell lines revealed that extracellular SF was rapidly depleted in the medium and that intracellular enrichment of SF was driven by conjugation of SF to GSH. Initially, the reversible conjugation of SF to GSH was found to be faster than SF binding to proteins. However, after 4 h of incubation, proteins became the major targets of SF while intracellular levels of the SF-GSH conjugate declined (Mi et al., 2007, 2011; Nakamura et al., 2018). This is because the resulting SF-GSH conjugates are still biologically active metabolites that modify protein thiols through transthiocarbamoylation (Shibata et al., 2011). We hypothesize that proteins are also the major targets of SF in plants. Untargeted proteomics has been used to identify – thus far – more than 30 SF-modified proteins in animals (reviewed in Mi et al., 2011). For instance, tubulin was identified as an abundant SF-modified protein in animal cells and ITCs have been shown to disrupt the tubulin network leading to cell growth inhibition and cell death both in animal (Xiao et al., 2012) and plant cells (Overby et al., 2015a). Although many proteins are affected by SF in animals, only the Kelch-like ECH-associated protein 1 (KEAP1) – Nuclear factor erythroid 2-related factor 2 (NRF2) signaling system that triggers cell survival responses to endogenous and exogenous stressors can be considered a validated target at this time (Dinkova-Kostova et al., 2017). It is still unclear how SF or the SF-GSH conjugates induce gene activation in Arabidopsis. We propose that the mechanism likely involves covalent modification of yet unkown proteins.

To this end, we note that structurally different electrophiles induce a similar subset of genes likely through common signaling mechanisms. SF, BITC, AITC, and PGA_1_ share 42 induced genes, of which 71% are classified as heat responsive (Supplementary Data, Table S3). However, a rather high number of genes induced by each electrophile species was not induced by any of the three other electrophiles: AITC (1970, 85%), SF (125, 22%), BITC (10, 14%), and PGA_1_ (143, 29%) suggesting that structural features other than the thiol reactivity have a strong impact on gene regulation. RES species-specific responses can be caused by different absorption and elimination kinetics, structure specific affinities to target proteins and steric properties that can have a strong impact on covalent protein modification/signaling (Mueller and Berger, 2009).

Wound-responsive genes have been shown to be regulated predominantly through the jasmonate pathway (Devoto et al., 2005; Masuda et al., 2014). In addition, at least in Arabidopsis, we show that a subset of wound-induced genes including heat shock genes is regulated by ITCs (Figure 3). It has been shown that jasmonates can diffuse radially over at least eight cell layers from the producer cell (Masuda et al., 2014). We do not know how far isothiocyanates diffuse into intact tissues surrounding the damage but the distance is likely short due to the high reactivity and rapid metabolism of isothiocyanates. This suggests that only a few cell layers will get in contact with isothiocyanates that are released into the apoplastic space after wounding. The calculated partition coefficient (xLogP) values of jasmonic acid (xLogP 1.6) and sulforaphane (xLogP 1.4) as well as the other relevant physicochemical parameters are very similar. From the physicochemical parameters a good membrane permeability could be predicted for both compounds.

We determined the concentration of sulforaphane (SF) in the whole leave although only a small area of the leaves was wounded. Since SF is released at the wound site only, the average leaf concentration is much lower than the actual concentration at the wound site. After complete mechanical tissue disruption, about 12 μmol aliphatic and 3 μmol indolic glucosinolates per gram fresh weight (Barth and Jander, 2006) were shown to be degraded within 1 min leading to a corresponding local concentration of breakdown products in damaged tissues of about 15 mM. Assuming that about 90% of the breakdown products are nitriles (Wittstock et al., 2016) the remaining 10% isothiocyanates still correspond to a local concentration of 1500 μM. This exceeds the detoxification capacity of neighboring cells by far but will be diluted by radial diffusion into intact tissues. Since we did not have the spatial resolution to measure local concentrations *in vivo*, it remains to be clarified how deep endogenously produced ITCs can penetrate into intact tissues.

After infection with *Pseudomonas syringae* pv *tomato* DC3000:AvrRpm1, excessive SF formation promotes cell death in cells adjacent to the infection site. In line with this, different mutants impaired in pathogen-induced accumulation of SF displayed attenuated programmed cell death upon bacterial and oomycete effector recognition as well as decreased resistance to the fungal pathogen *Hyaloperonospora arabidopsidis* (Andersson et al., 2015). In addition to its toxic and cell death promoting effects at high local concentrations, SF can also act as a defense priming metabolite. SF provokes covalent modification of histone H3 in the promoter and promoter-proximal region of defense genes such as *WRKY6* and *PDF1*.*2* coinciding with chromatin unpacking, primed *WRKY6* expression, unprimed plant defensin *PDF1*.*2* activation, and reduced susceptibility to *Hyaloperonospora arabidopsidis* (Schillheim et al., 2018). Hence, subtoxic amounts of SF can diffuse into unaffected tissues to prime and trigger defense genes to confer protection against pathogens in naïve tissue. However, it is not clear which ITC-responsive genes or ITC-modified proteins are critical for pathogen defense. Besides their role in pathogen defense, ITCs may induce genes and/or directly modulate protein activities that prevent damage by ITCs. Indeed, pretreatment of seedlings with subtoxic doses of SF and BITC increased the resistance of the seedlings toward the model ITC BITC (Figure 7).

After application of a low, subtoxic dose of SF, BITC, or the oxylipin RES PGA_1_ to seedlings, the most strongly induced genes were HSFA1-dependent heat-responsive genes (Table 1). Moreover, it has been shown that pretreatment of seedlings with high ITC concentrations (>1 mM, spray application) confers thermotolerance (Hara et al., 2013). Besides heat stress, it has been suggested that the heat-shock/chaperone network protects against multiple stresses associated with protein misfolding and aggregation (Farmer and Mueller, 2013; Jacob et al., 2017). We found that *hsfA1* Arabidopsis seedlings displayed higher sensitivity toward ITCs (Figure 6). However, over-accumulation of HSPs by heat acclimation did not increase the resistance toward ITCs suggesting that the heat shock-like response toward ITCs in the wild type could be sufficient to provide some level of protection against chemical proteotoxicity. Alternatively, the higher sensitivity of the *hsfA1* mutant line could be due to pleiotropic effects caused by constitutive lower expression of HSFA1-regulated genes in the mutant (Yoshida et al., 2011).

Besides heat shock genes, ITC as well as structurally unrelated RES induce a battery of stress and detoxification genes including UDP-glucosyl transferases as well as GSH S-transferases (GSTs) that are thought to play an important role in ITC and RES detoxification (Wagner et al., 2002). Although SF induced 10 GSTs more than 2-fold in our transcriptome analysis and AITC was reported to induce at least 13 GSTs more than 1.6-fold (Overby et al., 2015b) BITC did not induce any GST in our transcriptomic analysis (Supplementary Data, Table S4). Moreover, the endogenous GSH pool is far too low to detoxify exogenous SF completely suggesting that reversible GSH conjugation is an inefficient detoxification mechanism in the presence of high ITC concentrations.

Potentially other genes that are induced by SF, BITC, AITC, and PGA_1_ may play a more important role in increasing resistance to ITC or RES. However, out of the 42 genes that were found to be induced by all the RES (Supplementary Data, Table S3), most genes are HSFA1-dependent heat shock genes. Alternatively, induced resistance toward ITCs is due to altered post-transcriptional regulation mechanisms.

To conclude, we provide evidence that exposure of undamaged plant tissues to isothiocyanates induces host protection mechanisms that protect against intoxication by products of the mustard oil bomb. We hypothesize that this mechanism may reduce the collateral damage in intact tissues after wound-induced release of toxic isothiocyanates.

## Materials and Methods

### Plant Growth Conditions and Chemicals

*Arabidopsis thaliana* wild-type ecotypes “Columbia” (Col-0) and “Wassilewskija” (WS) as well as the mutant lines *hsfA1 abde* (provided by K. Yamaguchi-Shinozaki; Yoshida et al., 2011), *hsfA2* (SALK_008978, characterized by Charng et al., 2007) *tgg1 tgg2* (provided by G. Jander; Barth and Jander, 2006) were grown in a growth chamber under an 8 h/16 h short-day cycle at 22°C (80 μE)/20°C and either grown on soil or in liquid medium in 24-well plates. For liquid culture, seedlings (10 seeds per well) were grown in 500 μl of sterile MES buffered Murashige and Skoog (MS) medium, pH 5,7 (Duchefa Biochemie BV, Haarlem, Netherlands) supplemented with 3% sucrose for 7 days on a rotary shaker (100 rounds per minute). Thereafter, the medium was replaced by fresh medium with 3% sucrose and experiments were performed on day 10. The average weight of 10 seedlings was 123 ± 33 mg FW, experiments were performed biologically independent at least in triplicate (each replicate comprised 10 seedlings, see figure legends). For the isothiocyanate toxicity and chemical priming experiments the medium was replaced by liquid MS medium without sucrose on day 7 and at least three biologically independent experiments were performed on day 10. Wounding experiments were performed with plants (6 weeks old) grown on soil by wounding leaves three times with a forceps (4 mm broad) across the leaf lamina (90% angle to the midrib). Sulforaphane, radicicol and PGA_1_ were purchased from Cayman Chemical (Ann Arbor, United States). Benzyl isothiocyanate was from Merck KGaA (Darmstadt, Germany). DL-[D_8_]sulforaphane was obtained from Lipidox (Stockholm, Sweden). All solvents were at least HPLC grade and were purchased from Biosolve (Valkenswaard, Netherlands).

### Chemical Treatments

The chemicals were freshly dissolved in methanol and the stock solution was diluted into liquid MS medium without sucrose to yield the final treatment solution. The final methanol concentration was 1% (v/v) in all experiments except the chemical priming experiments. In these experiments (Figure 7), the methanol concentration during the pretreatment, the high dose treatment and recovery was 1, 2, and 0%, respectively. For controls, treatment solutions without chemicals but the same methanol concentrations were prepared. Ten seedlings (10-day-old) per well were exposed to the chemicals by replacing the medium with the treatment or control solution (500 μl). For recovery, treatment solutions were replaced by MS medium without sucrose at the times indicated and cultivated for additional 7 days. Seedlings were considered to be dead when the cotelydons and true leaves were completely bleached by the treatment.

### Gene Expression Analysis

Extraction of total RNA from plant material (ten 10-day-old-seedlings/sample; three biological replicates in one experiment) was performed by using peqGOLD TriFast^TM^ reagent (PEQLAB). RNA concentration was determined spectrophotometrically. Remaining DNA was removed using RNase-free DNase I (Fermentas, Waltham, United States). RNA was reverse transcribed to cDNA using RNA M-MLV reverse transcriptase (Promega, Madison, United States). Real-time PCR was performed using ABsolute SYBR Capillary Mix (Thermo Fisher Scientific, Waltham, United States) and a CFX 96 Real-Time System C1000 Thermal Cycler (Bio-Rad, Hercules, United States). Sequences of primers (TIB MOLBIOL, Berlin, Germany) are given in Supplementary Data, Table S5. The efficiency of the reaction for each primer pair was monitored by testing sequential dilutions of a preparation of the product with a defined concentration. The amplified fragment of each primer pair was sequenced. The annealing temperature for all primers was 59°C. Gene expression relative to *AtSAND* or *ACTIN 2/8* was measured by using the delta cycle threshold method (Pfaffl, 2001). *ACTIN* is not regulated by heat (Li et al., 2019). Our microarray studies revealed that both *ACTIN* and *SAND* are not regulated by isothiocyanates and appear to be suitable reference genes.

### Microarray Hybridization and Analysis

For transcriptome profiling, three biological replicates within one experiment samples were hybridized on an Agilent Platform using the Agilent Arabidopsis V4 (design number 021169) microarrays^1^. RNA quantity was measured with a ND-100 Spectrophotometer v3.3.0 (NanoDrop Technologies). RNA integrity was confirmed using an Agilent RNA 6000 Nano Chip on an Agilent 2100 BioAnalyzer (vB.02.03 BSI307). Total RNA (200 ng) was used for each sample labeling. Labeling and preparation of samples for hybridization was performed as described in the one-color microarray-based gene expression analysis protocol provided by Agilent including the one-color RNA spike-in kit (v5.0.1, 2006; Agilent Technologies, Santa Clara, united States). Slides were scanned on the Agilent Microarray Scanner with extended dynamic range (XDR) at high resolution (5 μm). Data sets were extracted by feature extraction software package (v11.5.1.1/Agilent Technologies) using a standard protocol (GE1_1105_Oct12).

Data preprocessing was performed using the Bioconductor software (Huber et al., 2015) with the statistical programming environment R (R Development Core). Normalization has been performed using negative control probes and quantile normalization using negative and positive control probes as implemented in the neqc function (Shi et al., 2010) of the Limma package (Ritchie et al., 2015). Differential gene expression for all stimuli was calculated using the moderated t-statistic approach as implemented in the R-package Limma, which has been specifically developed for the analysis of small sample size experiments. The *P*-values of all results were corrected for multiple testing by using the false discovery rate (FDR) (Benjamini and Hochberg, 1995). The data discussed in this publication has been deposited in NCBI’s Gene Expression Omnibus (Edgar et al., 2002) and are accessible through GEO Series accession number GSE117869^2^.

### Analysis of SF

Seedlings (100 mg, 10 seedlings/sample) or leaves (100 mg) were shock-frozen in liquid nitrogen and incubated with 500 μl of methanol/water/formic acid (9:1:0.1, v/v) at 80°C for 1 min. DL-[D_8_]SF (50 pmol) was added as internal standard. Biological replicates within one experiment were performed in triplicate. SF was extracted using a ball mill (Retsch Inc., Germany) operated at 23 Hz for 2 min. After centrifugation (10 min at 10,000 *g*), the supernatant was stored at −20°C until analysis. SF was analyzed with an Acquity Ultra Performance Liquid Chromatography system (UPLC) coupled to a triple quadrupole mass spectrometer (Quatro Premier, Waters, Milford, MA, United States). Chromatographic separation was carried out on a BEH C18 column (2.1 × 50 mm, 1.7 μm, Waters) with a linear binary solvent gradient of 5–60% eluent B over 5 min at a flow rate of 0.2 mL min^–1^. Eluent A consisted of 1 mM ammonium acetate in water and eluent B was methanol.

SF was detected by Multiple Reaction Monitoring (MRM) in the positive electrospray mode with a capillary voltage of 2.75 kV. Argon was used for collision-induced dissociation (CID) (flow rate of 0.3 mL min^–1^, 3 × 10^–3^ mBar). The cone voltage and collision energy were 32 V and 16 V, respectively. The following ions were recorded (m/z precursor ion, m/z product ion): SF (178, 114), [D_8_]SF (186, 122).

### Quantification of GSH, Glutathione Disulfide (GSSG), and SF Glutathione (SF-GSH) Conjugate

GSH, GSSG, and SF-GSH were extracted from 100 mg of seedlings (10 seedlings/sample) with 500 μl of methanol/water/formic acid (9:1:0.1, v/v) containing 10 nmol glutathione ethylester (GSH-EE internal standard) and 8.5 mM S-methyl methanethiosulfonate (for conversion of the thiol groups of GSH and GSH-EE into dithiomethanes) at 80°C for 1 min. Biological replicates within one experiment were performed in triplicate. Analytes were extracted using a ball mill (23 Hz, 2 min) and the homogenizate was centrifugated (10 min at 10,000 *g*). The supernatants were analyzed using an Acquity UPLC coupled to a quadrupole/time-of-flight mass spectrometer (qTOF-MS, Synapt G2 HDMS, Waters, Milford, MA, United States). Chromatographic separation was carried out on a BEH C18 column (2.1 × 100 mm, 1.7 μm, Waters) with a linear binary solvent gradient of 0–60% eluent B over 5 min at a flow rate of 0.3 mL min^–1^. Eluent A consisted of 0.1% formic acid in water and eluent B was methanol. The mass spectrometer was operated in the positive electrospray ionization mode. Peak areas were integrated in the extracted ion chromatograms of GSH dithiomethane (m/z of 354.074 ± 0.03, retention time of 2.9 min), GSSG (m/z of 613.160 ± 0.03, retention time of 2.0 min), SF-GSH (m/z of 485.112 ± 0.03, retention time of 3.1 min) and GSH-EE dithiomethane (m/z of 382.110 ± 0.030, retention time of 4.1 min). For analyte quantification, response factors were determined from calibration curves using authentic reference compounds (GSH, GSSG, and GSH-EE). SF-GSH was synthetized by incubation of SF with GSH (molar ratio of 1:10) in a 1:1 (*v*/*v*) mixture of methanol and phosphate buffer (50 mM, pH 8.0) at room temperature for 10 min (remaining SF < 2%). The reaction was terminated by acidifying the sample with formic acid to pH 4. SF-GSH was stable for at least 24 h at 10°C and 5 days at −20°C.

### Measurement of Chlorophyll Fluorescence

Pulse amplitude modulation fluorometry (PAM) was used to measure chlorophyll fluorescence in seedlings. Chlorophyll fluorescence was measured with a Maxi Imaging PAM Chlorophyll Fluorometer (Walz GmbH, Germany) using the saturation pulse method as described (Schreiber, 2004; Bonfig et al., 2006). Seedlings were dark adapted for 10 min prior to the measurements. The optimal quantum yield of PSII (*F*_*v*_/*F*_*m*_) was determined using the software ImagingWin version 2.41a (Walz GmbH) as described (Van Kooten and Snel, 1990).

## Data Availability Statement

The datasets generated for this study can be found in the NCBI’s Gene Expression Omnibus under accession number GSE117869 (https://www.ncbi.nlm.nih.gov/geo/query/acc.cgi?acc=GSE117869).

## Author Contributions

SB, AF, and MM: conceptualization. MK: developed the methodology. EF, JG, MS, MK, and AF: performed the experiments. EF, MS, MK, MD, TM, and SB: data analysis and formal analysis. MM: wrote the original draft, funding acquisition, and supervision. SB and MM: reviewing and editing. All authors contributed to the article and approved the submitted version.

## Conflict of Interest

The authors declare that the research was conducted in the absence of any commercial or financial relationships that could be construed as a potential conflict of interest.

## Footnotes

1 http://www.agilent.com

2 http://www.ncbi.nlm.nih.gov/geo/query/acc.cgi?acc=GSE117869

## Abbreviations

AITC: allyl isothiocyanate
BITC: benzyl isothiocyanate
BSO: buthionine sulfoximine
GSH: glutathione
GST: glutathione S-transferase
GSSG: glutathione disulfide
HSF: heat shock factor
HSP: heat shock protein
ITC: isothiocyanate
PGA_1_: prostaglandin A_1_
RES: reactive electrophilic species
SF: sulforaphane
TGG: ß-thioglucoside glucohydrolases.
