# Supplementary material for: Chemical Priming by Isothiocyanates Protects Against Intoxication by Products of the Mustard Oil Bomb
Source: Front Plant Sci. 2020 Jun 26;11:887. doi: 10.3389/fpls.2020.00887 (PMC7333730; doi:10.3389/fpls.2020.00887)
Supplement: Supplementary file 1 [file Data_Sheet_1.docx]

Supplementary Material

# Supplementary Data

This file includes:

Supplemental Figures S1 and S2

Supplemental Table S1, S3, S4 and S5

Other supplemental data items for this manuscript include the following:

Supplemental Table S2 (Excel spreadsheets)

# Supplementary Figures and Tables

## Supplementary Figure S1

**Supplementary Figure 1**. Venn diagram comparing regulated genes by SF, BITC, PGA_1_ and AITC. Overlaps of genes over-expressed (fold change ≥ 2), A, or under-expressed (fold change ≤ 0.5), B, after treatments of Arabidopsis seedlings with SF (100 µM), BITC (100 µM) and PGA_1_ (75 µM) for 4 h (Suppl. Table S2) or exposure to AITC vapour for 9 h (Kissen et al., 2016).

## Supplementary Figure S2

**Supplementary Figure 2.** Heat priming without effect on resistance towards BITC. Arabidopsis seedlings grown in liquid culture in MS medium were grown at 22°C (control, white bars) or heat acclimated at 37° for 2 h (black bars) followed by 2 h of recovery at 22°C. Thereafter, seedlings were treated with BITC at the concentration indicated for 2 h and, subsequently, allowed to recover in MS medium. The effect of the treatment combination on photosynthesis (F_v_/F_m_) was determined after 4 h of recovery in dark-adapted leaves. Data represent means ± SE, n=6. Survival rates were determined 7 days after the treatments (n = 52-69).

## Supplementary Table S1

**Supplementary Table S1.** List of metabolites whose abundance increased significantly in Arabidopsis seedlings 4 h after SF application. Metabolite fingerprinting was performed to identify metabolites of SF. Methanolic extracts were analysed with UPLC-qTOF-MS by applying positive and negative ESI modes. The chromatogratographic conditions were the same as used for quantification of SF. ANOVA was applied to filter out peaks whose levels were significantly higher in the treated seedlings compared to the controls (p≤0.05, fold change ≥ 5, signal-to-noise ratio ≥ 10, n = 3). Differing peaks were manually inspected and verified in an independently performed experiment. Out of 1053 annotated peaks (positive ESI: 535, negative ESI: 518), 8 peaks (positive ESI: 5, negative ESI: 3) were identified. Raphasunamic acid could be identified in addition to SF and SF-GSH utilising exact mass (error ≤1 mDa), isotope abundance distribution and fragment pattern. Structures of the other two differing peaks could not be elucidated due to their low intensity.

| **Identified metabolite** | **Anova (p)** | **Fold Change** | **Ion** | **ESI** | **m/z** | **RT (min)** |
| --- | --- | --- | --- | --- | --- | --- |
| sulphoraphan-glutathion | 1,04E-12 | Infinity | [M+H]^+^ | positive | 485,120 | 2,12 |
|  | 3,39E-10 | Infinity | [M+Na]^+^ | positive | 507,101 | 2,13 |
|  | 2,51E-08 | Infinity | [M-C_6_H_12_NOS_2_-H]^-^ | negative | 306,076 | 2,13 |
| sulphoraphan | 1,70E-03 | 5,2 | [M+H]^+^ | positive | 178,036 | 3,94 |
|  | 5,27E-03 | 4,8 | [M-CH_3_OS+H]^+^ | positive | 114,037 | 3,94 |
| raphasunamic acid | 1,76E-02 | 14,7 | [M-H]^+^ | negative | 161,968 | 1,07 |
| unknown | 4,28E-06 | Infinity |  | negative | 296,009 | 2,36 |
| unknown | 1,12E-04 | 1941,0 |  | positive | 103,057 | 2,46 |

## Supplementary Table S3

**Supplementary Table S3.** List of the 42 common genes up-regulated by SF, BITC and PGA_1_. Regulated genes after treatment for 4 h with SF, BITC and PGA_1_ (Suppl. Table 2) as well as treatment with AITC for 9 h (Kissen et al., 2016). Only genes with a fold change ≥ 2 and an adjusted p-value ≤ 0.05 were considered.

| **Locus** | **Description** |
| --- | --- |
| AT4G27670 | HSP21 (heat shock protein 21); chloroplast located small heat shock protein. |
| AT1G53540 | HSP20-like chaperones superfamily protein |
| AT1G52560 | HSP20-like chaperones superfamily protein |
| AT3G09640 | APX1B (ASCORBATE PEROXIDASE 1B); Encodes a cytosolic ascorbate peroxidase APX2. Ascorbate peroxidases are enzymes that scavenge hydrogen peroxide |
| AT4G25200 | ATHSP23.6-MITO (mitochondrion-localized small heat shock protein 23.6); |
| AT5G12020 | HSP17.6II (17.6 kDa class II heat shock protein) |
| AT4G10250 | ATHSP22.0 (HSP20-like chaperones superfamily protein); Columbia endomembrane-localized small heat shock protein |
| AT3G46230 | ATHSP17.4 (ARABIDOPSIS THALIANA HEAT SHOCK PROTEIN 17.4); member of the class I small heat-shock protein (sHSP) family, which accounts for the majority of sHSPs in maturing seeds |
| AT5G12030 | AT-HSP17.6A (heat shock protein 17.6A); Encodes a cytosolic small heat shock protein with chaperone activity that is induced by heat and osmotic stress and is also expressed late in seed development. |
| AT2G26150 | ATHSFA2 (heat shock transcription factor A2); member of heat stress transcription factor (Hsf) family. Involved in response to misfolded protein accumulation in the cytosol. Regulated by alternative splicing and non-sense-mediated decay. |
| AT1G07400 | HSP20-like chaperones superfamily protein |
| AT1G59860 | HSP20-like chaperones superfamily protein |
| AT1G03070 | Bax inhibitor-1 family protein |
| AT1G72660 | P-loop containing nucleoside triphosphate hydrolases superfamily protein |
| AT5G05220 | unknown protein |
| AT2G29500 | HSP20-like chaperones superfamily protein |
| AT1G07500 | unknown protein |
| AT1G44414 | unknown protein |
| AT2G32120 | HSP70T-2 (heat-shock protein 70T-2) |
| AT1G54050 | HSP20-like chaperones superfamily protein |
| AT1G16030 | Hsp70b (heat shock protein 70B) |
| AT4G12400 | stress-inducible protein, putative |
| AT4G25380 | SAP10 (stress-associated protein 10) |
| AT5G59720 | HSP18.2 (heat shock protein 18.2); encodes a low molecular weight heat shock protein that contains the heat shock element in the promoter region. Expression is induced in response to heat shock. |
| AT5G07330 | unknown protein |
| AT4G21320 | HSA32 (HEAT-STRESS-ASSOCIATED 32); Encodes heat-stress-associated 32-kD protein. Up-regulated by heat shock. Thermotolerance in a knockout mutant was compromised following a long recovery period (> 24 h) after acclimation heat shock treatment. |
| AT3G12580 | ATHSP70 (ARABIDOPSIS HEAT SHOCK PROTEIN 70) |
| AT2G47180 | ATGolS1 (Galactinol synthase 1) |
| AT1G74310 | ATHSP101 (heat shock protein 101); encodes ClpB1, which belongs to the casein lytic proteinase/heat shock protein 100 (Clp/Hsp100) family. Involved in refolding of proteins which form aggregates under heat stress. Also known as AtHsp101. AtHsp101 is a cytosolic heat shock protein required for acclimation to high temperature. |
| AT3G17400 | F-box family protein |
| AT2G46240 | ATBAG6 (ARABIDOPSIS THALIANA BCL-2-ASSOCIATED ATHANOGENE 6); A member of Arabidopsis BAG (Bcl-2-associated athanogene) proteins, plant homologs of mammalian regulators of apoptosis. Expression of BAG6 in leaves was strongly induced by heat stress. Knockout mutants exhibited enhanced susceptibility to fungal pathogen Botrytis cinerea. Plant BAG proteins are multi-functional and remarkably similar to their animal counterparts, as they regulate apoptotic-like processes ranging from pathogen attack, to abiotic stress, to plant development. The mRNA is cell-to-cell mobile. |
| AT3G60140 | BGLU30 (BETA GLUCOSIDASE 30); Encodes a protein similar to beta-glucosidase and is a member of glycoside hydrolase family 1. Expression is induced after 24 hours of dark treatment, in senescing leaves and treatment with exogenous photosynthesis inhibitor. Induction of gene expression was suppressed in excised leaves supplied with sugar. The authors suggest that the gene's expression pattern is responding to the level of sugar in the cell. The mRNA is cell-to-cell mobile. |
| AT1G48700 | 2-oxoGlutarate (2OG) and Fe(II)-dependent oxygenase superfamily protein |
| AT1G60750 | NAD(P)-linked oxidoreductase superfamily protein |
| AT2G47770 | ATTSPO (TSPO(outer membrane tryptophan-rich sensory protein)-related); Encodes a membrane-bound protein designated ATTSPO (Arabidopsis Thaliana TSPO-related). |
| AT5G64750 | ABR1 (ABA REPRESSOR1); Encodes a putative transcription factor containing an AP2 domain. Is a member of the ERF (ethylene response factor) subfamily B-4 |
| AT4G39360 | unknown protein |
| AT1G07985 | Expressed protein |
| AT1G62570 | FMO GS-OX4 (flavin-monooxygenase glucosinolate S-oxygenase 4); belongs to the flavin-monooxygenase (FMO) family, encodes a glucosinolate S-oxygenase that catalyzes the conversion of methylthioalkyl glucosinolates to methylsulfinylalkyl glucosinolates The mRNA is cell-to-cell mobile. |
| AT1G69600 | ATHB29 (ARABIDOPSIS THALIANA HOMEOBOX PROTEIN 29); Encodes ZFHD1, a member of the zinc finger homeodomain transcriptional factor family. Binds to the 62 bp promoter region of ERD1 (early responsive to dehydration stress 1). Expression of ZFHD1 is induced by drought, high salinity and abscisic acid. |
| AT5G01380 | Homeodomain-like superfamily protein |
| AT2G17500 | PILS5, PIN-LIKES 5;Auxin efflux carrier family protein |

## Supplementary Table S4

**Supplementary Table S4** Regulation of genes of the glutathione S-transferase family by ITCs and PGA_1_. Regulation of GSTs with a fold change ≥ 2 (dark grey) or ≤ 0.5 (light grey) and an adjusted p-value ≤ 0.05 after treatment for 4 h with SF, BITC, PGA_1_ (Suppl. Table 2) or AITC for 9 h (Kissen et al., 2016) is shown.

| **Protein Name:** |  | **Sf** | **BITC** | **PGA_1_** | **AITC** |
| --- | --- | --- | --- | --- | --- |
|  |  | **FC** | **FC** | **FC** | **FC** |
| **Phi family** |  |  |  |  |  |
| AtGSTF2 (GST4) | AT4g02520 |  |  |  |  |
| AtGSTF3 | At2g02930 |  |  |  |  |
| AtGSTF4 | AT1g02950 |  |  |  |  |
| AtGSTF5 | AT1g02940 |  |  |  |  |
| AtGSTF6 (GST1) | AT1g02930 |  |  |  | **3,50** |
| AtGSTF7 | AT1g02920 |  |  |  | **4,44** |
| AtGSTF8 (GST6) | AT2g47730 | **2,98** |  | **3,06** | **4,51** |
| AtGSTF9 | AT2g30860 |  |  |  | 0,41 |
| AtGSTF10 | AT2g30870 |  |  |  |  |
| AtGSTF11 | AT3g03190 | 0,44 | 0,43 |  | 0,11 |
| AtGSTF12 | AT5g17220 |  |  |  |  |
| AtGSTF13 | AT3g62760 |  |  | 0,43 | **4,52** |
| AtGSTF14 | AT1g49860 | 0,10 | 0,15 | 0,12 | 0,37 |
|  |  |  |  |  |  |
| **Tau family** |  |  |  |  |  |
| AtGSTU1 | AT2g29490 |  |  |  | **3,94** |
| AtGSTU2 | AT2g29480 | **5,68** |  | **3,55** | **11,00** |
| AtGSTU3 | AT2g29470 | **8,41** |  | **5,72** | **3,31** |
| AtGSTU4 | AT2g29460 | **5,77** |  | **3,33** | **8,22** |
| AtGSTU5 | AT2g29450 |  |  |  | **8,19** |
| AtGSTU6 | AT2g29440 |  |  |  |  |
| AtGSTU7 | AT2g29420 | **2,03** |  | **2,94** | **3,05** |
| AtGSTU8 | AT3g09270 |  |  |  |  |
| AtGSTU9 | AT5g62480 |  |  |  | **2,08** |
| AtGSTU10 | AT1g74590 | **2,03** |  |  | **3,40** |
| AtGSTU11 | AT1g69930 |  |  |  |  |
| AtGSTU12 | AT1g69920 | **2,60** |  | **2,22** | **4,12** |
| AtGSTU13 | AT1g27130 |  |  |  |  |
| AtGSTU14 | AT1g27140 | 0,08 | 0,24 | 0,34 |  |
| AtGSTU15 | AT1g59670 |  |  |  |  |
| AtGSTU16 | AT1g59700 |  |  |  | **2,02** |
| AtGSTU17 | AT1g10370 |  |  |  | **4,28** |
| AtGSTU18 | AT1g10360 |  |  |  |  |
| AtGSTU19 | AT1g78380 |  |  | **2,32** | **3,44** |
| AtGSTU20 | AT1g78370 |  |  |  | **0,49** |
| AtGSTU21 | AT1g78360 |  |  |  |  |
| AtGSTU22 | AT1g78340 | **3,75** |  | **3,36** | **2,20** |
| AtGSTU23 | AT1g78320 |  |  |  |  |
| AtGSTU24 | AT1g17170 | **3,28** |  | **3,33** | **9,61** |
| AtGSTU25 | AT1g17180 | **3,73** |  | **2,90** | **7,87** |
| AtGSTU26 | AT1g17190 |  |  |  |  |
| AtGSTU27 | AT3g43800 |  |  |  |  |
| AtGSTU28 | AT1g53680 | **0,39** |  |  |  |
|  |  |  |  |  |  |
| **Zeta family** |  |  |  |  |  |
| AtGSTZ1 | AT2g02390 |  |  |  |  |
| AtGSTZ2 | AT2g02380 |  |  |  |  |
|  |  |  |  |  |  |
| **Theta family** |  |  |  |  |  |
| AtGSTT1 | AT5g41210 |  |  |  |  |
| AtGSTT2 | AT5g41240 |  |  |  |  |
|  |  |  |  |  |  |
| **Lambda family** |  |  |  |  |  |
| AtGSTL1 | AT5g02780 |  |  |  | **4,96** |
| AtGSTL2 | AT3g55040 |  |  |  |  |
| AtGSTL3 | AT5g02790 |  |  |  |  |
|  |  |  |  |  |  |
| **Dehydroascorbate reductase family** | |  |  |  |  |
| AtDHAR1 | AT1g19570 |  |  |  |  |
| AtDHAR2 | AT1g75270 |  |  | **2,11** | **8,26** |
| AtDHAR3 | AT5g16710 |  |  |  |  |
| AtDHAR4 | AT5g36270 | **0,32** |  |  |  |
| **Tetrachlorohydroquinone dehalogenase-like family** | | |  |  |  |
| AtTCHQD1 | AT1g77290 |  |  |  |  |

## Supplementary Table S5

**Supplementary Table S5.** List of primers and accession numbers for genes used in this study.

*List of primers*

| **Gene** | **Forward primer** | **Reverse primer** |
| --- | --- | --- |
| *HSP101*  (At1G74310) | 5′-TGAGCTAGCTGTGAATGCAG-3′ | 5′-TCAACTGGTCAACAGCCAAA-3′ |
| *HSFA2*  (AT2G26150) | 5`-CAGCAAGGATCTGGGATGTC-3` | 5`- GCTGTTGCCTCAACCTAACT-3` |
| *DREB2A*  (At5g05410) | 5`-AGGGTCGAAGAAGGGTTGTA-3` | 5`-CAGCTTCTTGAGCAGTAGGG-3` |
| *HSP26.5*  (At1g52560) | 5`-TGTGAAAGAGGTTTGGTCGG-3` | 5`-TGTTACGCCAGAGGCTTTTT-3´ |
| *HSP17.6II*  (AT5G12020) | 5’-AAGTCCCCGAAGACCACAAC-3’ | 5’-CGATGACGTCAGCAGGTGTA-3’ |
| *Cyp81D11*  (AT3G28740) | 5’-ATTGCCGAGGTAGTTGT-3’ | 5’-TTGCCTTTCGTAATACT-3’ |
| *AtSAND*  (At2g28390) | 5′-AACTCTATGCAGCATT-3′ | 5′-GGTGGTACTAGCACAA-3′ |
| *ACTIN 2/8*  (At5g09810, At1g49240) | 5′-GGTGATGGTGTGTCT-3′ | 5′-ACTGAGCACAATGTTAC-3′ |

*Accession Numbers*

The following genes referred to in the text are listed with their accession numbers: *TGG1*, AT5G26000; *TGG2*, AT5G25980; *HSFA2*, AT2G26150; *HSP17.6II*, AT5G12020; *HSP26.5*, At1g52560; *HSP101*, AT1G74310; *DREB2A*, AT5G05410; *CYP81D11*, AT3G28740; *TH8*, AT1G69880; *UGT74E2*, AT1G05680.
